# Unsteady MHD free convection flow of an exothermic fluid in a convectively heated vertical channel filled with porous medium

**DOI:** 10.1038/s41598-022-16064-y

**Published:** 2022-07-14

**Authors:** Muhammed Murtala Hamza, Abdulsalam Shuaibu, Ahmad Samaila Kamba

**Affiliations:** 1 Department of Mathematics, Usmanu Danfodiyo University Sokoto, P.M.B 2346, Sokoto, Nigeria; 2 Department of Mathematics, Federal University of Agriculture Zuru, P.M.B 28, Zuru, Nigeria

**Keywords:** Mathematics and computing, Applied mathematics

## Abstract

Utilizing porous media in a new mathematical model to improve convective heat transfer characteristics in a variety of applications, such as radiation nuclear disposal storing, evaporation cooling, sieving, geological extraction, crude petroleum refining, and building heating and cooling, is becoming increasingly important. This study proposed a numerical analysis of the unsteady magnetohydrodynamic free convection flow of an exothermic fluid with Newtonian heating. This discovery reveals two types of solutions: steady state and unsteady state. After transforming the governing equation from dimensional form to dimensionless form, the steady state governing equation was solved by the Homotopy Perturbation Method. However, the implicit finite difference approach is used to solve the time-dependent governing equations numerically. The impact of various emerging parameters, namely the Hartmann number, Boit number, Darcy number, Navier slip parameter, and the Frank-Kamenetskii parameter, was discussed and graphically analyzed. During the computations and analysis, it was discovered that a minor rise in the Hartman number results in the Lorentz force, which streamlines the momentum barrier layer and hence slows the fluid flow. The fluid velocity, on the other hand, rose as the porous medium, thermal Biot number, slip parameter, and temperature field increased as the viscous reactive fluid parameter and Newtonian heating increased. The skin friction and Nusselt number were also examined and reported. By comparing the finding to an existing work, a great agreement was revealed.

## Introduction

Magnetohydrodynamics has gotten a lot of interest lately because of its success in solving a variety of problems in various geometries. Mixed convection was studied by Jha and Aina^1^ in a microchannel with electrically isolated infinite vertical parallel walls at equal spacing in previous work. A numerical approach was used by Uddin et al.^2^ to determine the combined impact of Newtonian heating and radiation. By taking into account Newtonian heating and radiation effects, Das and his co-authors^3^ address their subject experimentally. The effects of Newtonian and joule heating on MHD flow were studied numerically by Chaudhary and his colleagues^4^. Research by Kumar et al.^5^ shows that natural convection flow is influenced by the Hartman number, which in turn effects the Hall effect. In order to better understand the effects of radiation, Jha et al.^6^ devised a computational method. Employing homotopy analysis a variety of scholars are working on different elements of hydromagnetic flow and convective heat transfer with thermal radiation. The following articles provide further information for those who are interested: As stated by Chamkha^7^, the problem of hydromagnetic, fully developed laminar mixed convection flow in a vertical channel with symmetric and asymmetric wall heating conditions As an alternative, the Hall and ion slip effects on the MHD convective flow of elastico-viscous fluid through a porous medium between two rigidly rotating parallel plates with a time-varying sinusoidal pressure gradient were studied by VeeraKrishna and Chamkha^8^. According to their result, fluid motion in a thinner boundary layer is hindered by elasticity and magnetic fields. When the pressure gradient oscillates at a lower frequency, it suppresses the reverse flow. There is an MHD flow over a moving plate in a rotating fluid with a magnetic field, Hall currents, and free stream velocity evaluated by Takhar et al.^9^. According to their observations, the skin friction coefficients for primary and secondary flows rise with an increase in the Coriolis force of the magnetic field, Hall currents, and wall velocity. An investigation of the effects of non-linear radiation on MHD flow was conducted by Khan and Mustafa^10^. Natural convection of viscous incompressible viscous reactive fluid was studied by Ojemeri et al. using a vertical porous cylinder with a radial magnetic field^11^. MHD flow with Newtonian heating is studied numerically by Ullah and his co-authors^12^. Chamkha^13^ investigates fluid-particle flow and heat transport in channels and circular pipes, which are solved in closed form using Fourier cosine and Bessel functions. Due to its importance in research and engineering, Daniel and Daniel^14^ investigated the effects of buoyancy and heat radiation on MHD flow. In their study of steady state MHD natural convection slip flow, Hamza et al.^15^ used a vertical geometry and found that increasing the chemical reaction value had no effect on fluid momentum. Extensive studies have been undertaken which involve such fluids^16–18^ According to Kumar and Singh^19^, MHD flow with convective heat heating and cooling and induced magnetic field may have been enthusiastically accepted because of its relevance in research and engineering.

Convective heat transfer characteristics can be improved by using porous media in a new mathematical model. This strategy is becoming increasingly important in a variety of applications, including radioactive nuclear waste storage, transpiration cooling, filtration, geothermal extraction, and crude oil extraction. In a microchannel filled with porous material, Gireesha and Sindhu^20^ investigated the MHD convection flow of casson fluid. The systems of subsurface water circulation, biological sciences, and other subjects was reported by Rashed et al.^21^. It was shown that a transverse magnetic field may impact mixed-convection events in a porous material with continuous heat flow over a semi-infinite permeable vertical plate by Chamkha^22^. For natural convection flow around an isothermal ball, Chamkha et al.^23^ modified boundary-layer issue. Interested researchers might follow Mahmoodi et al.^24^ to find relevant literature on porous media applications.

Turbulent boundary layer flow and heat transmission in a vertical channel filled with porous medium have evolved rapidly in recent years, with numerous real-world applications, such as the cleaning of inner joviality, the aortic root, and incorporated circuit and mechanical system processing methods. Innumerable scholars have contributed significantly to the understanding of fluid flow issues at the boundary layer by applying slip boundary conditions. For example, Zhu et al.^25^ used the Homotopy analysis technique to evaluate MHD slip flow around a centerline on a power law expanding strip. Wang^26^ investigated the impact of partial sliding on the expanded material. Empirical studies of an elastico-viscous fluid with partial slip were conducted by Ariel and colleagues^27^. Zhu et al.^28^ used Homotopy analysis to investigate axisymmetric slip stagnation point flow with a temperature jump in a relaxed state. There are st-udies by Chen^29,30^ in which the boundary layer flow and heat transfer characteristics on a level surface with no-slip boundary conditions were examined. The^31^ and^32^ discoveries may be tested on a vertical plate with slip flow and temperature jump boundaries. Makinde^33^ solved the problem of hydromagnetic heat and mass transport across a vertical plate with a convective surface boundary condition. MHD with Newtonian heating in vertical channels has been extensively studied in the literature since these findings were made. According to our findings, a thermal breakdown fluid in a convectively heated vertical channel filled with porous material experiences an unsteady MHD free convection flow, which has not been investigated to the best of our knowledge till now. This piece has a lot in common with Hamza's^32^. Natural convection MHD flow of an exothermic fluid in a vertical channel is studied in this paper. Analytical and numerical solutions were obtained using the implicit finite difference scheme approach and the Homotopy perturbation method. Study in the domains of glass fiber, wire drawing, papermaking, tarpaulin extraction, hot rolling, and drawing of plastic films can all benefit from the present research.

In terms of the study's uniqueness, it is not novel that in order to meet the increasing energy demands in a carbon-free global economy, it is necessary to develop novel and intelligent materials that can improve the efficiencies of energy conservation and energy conversion modules. Nevertheless, porous materials offer prospects for hydrocarbon and heat functionalities due to their unique properties in a wide range of real-world engineering fields, including agriculture, manmade cooling of soils, building, food and beverages, and storage applications. Magnetohydrodynamic (MHD) is the subfield of fluid dynamics in which magnetic fields play an essential role in fluid movement. Its existence in many configurations is a fascinating part of science and innovation, as well as real-world applications such as solar wind and flare, the planet's rotation fields, nuclear power, and science and medicine.

## Mathematical structure

Take into account the flow of an exothermic Arrhenius kinetic fluid with energy transfer and velocity slip in a channel formed by two endless vertical parallel force divided by a space H. The flow is produced by convective heating given to the lower surface of the channel wall, as well as the reactive nature of the fluid. Thus, according Hamza^32^ and Jha et al.^1^, the non-dimensional model equation under the Boussinesq's assumption can be stated as follows. The following is our presumption:

i. Thermal properties are considered constant
ii. The transverse magnetic field exerts a greater influence than the buoyancy force.
iii. Heat generation is neglected.
iv. Ignoring the effects of joule heating
v. The dissipation function is neglected; this is appropriate as long as the flow is not highly viscous or compressible.
  1 $$\frac{{\partial u^{\prime } }}{{\partial t^{\prime } }} = \nu \frac{{\partial^{2} u^{\prime } }}{{\partial y^{{^{\prime } 2}} }} - \frac{{\sigma B_{0} y_{0}^{2} }}{\rho \nu }u^{\prime } - \left( \frac{1}{k} \right)u^{\prime } + g\beta (T^{\prime } - T_{0}^{{^{\prime } }} )$$
  2 $$\frac{{\partial T^{\prime } }}{{\partial t^{\prime } }} = \frac{k}{{pC_{p} }}\frac{{\partial^{2} T^{\prime } }}{{\partial y^{\prime 2} }} + \frac{{QC_{0}^{*} A}}{{pC_{p} }}e^{{\left( {\frac{ - E}{{RT^{\prime } }}} \right)}}$$

For the current investigation, the following are the initial and boundary conditions:

3 $$\begin{aligned} & t^{\prime } \le 0:u^{\prime } 0,\;T^{\prime } \to T_{0}^{{^{\prime } }} ,\;0 \le y^{{^{\prime } }} \le H \\ & t^{\prime } 0:u^{\prime } = \gamma^{*} \frac{{\partial u^{\prime } }}{{\partial y^{{}} }},\; - k\frac{{\partial T^{\prime } }}{{\partial y^{\prime } }} = h[T_{0}^{{^{\prime } }} - T^{\prime } ],\;at\;y^{\prime } = 0 \\ & u^{\prime } = 0,\;T^{\prime } = T_{0}^{{^{\prime } }} ,\;as\;y^{\prime } \to H \\ \end{aligned}$$

Although $(\beta )$ is the heating value, $(Q)$ is reaction rate constants, kinetic energy and flowability are all represented by this term $(E)$ and $(A)$, initial concentration is represented by this term $(C_{0} )$. A fluid's thermal efficiency is often described as $(K)$, whereas its gravitational force is represented by $(g)$, its heat capacity at high pressure is expressed as $(C_{p} )$ and the fluid's strength is labeled by $(\rho )$.

The dimensionless variables and parameters shown below are used to solve Eqs. (1) and (2).

4 $$\begin{aligned} y & = \frac{{y^{\prime } }}{H},\;t^{\prime } = \frac{{t^{{^{\prime } }} \mu_{0} }}{{H^{2} }},\;\theta = \frac{{E(T^{{^{\prime } }} - T_{0} )}}{{RT_{0}^{2} }},\;\varepsilon = \frac{{RT_{0} }}{E},\;U = \frac{{u^{{^{\prime } }} \mu_{0} E}}{{g\beta H^{2} RT_{0}^{2} }},\;Gr = \frac{{g\beta (T_{1} - T_{0} )}}{{v^{2} }} \\ \lambda & = \frac{{QC_{0}^{*} AEH^{2} }}{{RT_{0}^{2} }}e^{{\left( {\frac{ - E}{{RT_{0} }}} \right)}} ,\;\Pr = \frac{{\mu_{0} pC_{p} }}{k},\;\gamma = \frac{{\gamma^{*} }}{H},\;\theta_{a} = \frac{{E(T_{a} - T_{0} )}}{{RT_{0}^{2} }},\;Br = \frac{hH}{k}, \\ Da & = \frac{{k_{0} }}{{b^{2} }},\;Ha^{2} = \frac{{\sigma B_{0}^{2} r_{0}^{2} }}{\rho \nu } \\ \end{aligned}$$

In Eqs. (1)–(3), use (4) and assume the following shape:

5 $$\frac{\partial u}{{\partial t}} = \frac{{\partial^{2} u}}{{\partial y^{2} }} - \left( {H^{2} a + \frac{1}{Da}} \right)U + \theta$$

6 $$\frac{{\partial^{2} \theta }}{\partial t} = \frac{1}{Pr}\frac{{\partial^{2} \theta }}{{\partial y^{2} }} + \frac{\lambda }{Pr}e^{{\frac{\theta }{1 + \varepsilon \theta }}}$$

The beginning and boundary conditions are given in non-dimensional form:

7 $$\begin{aligned} & U = 0,\;\theta = 0,\;0 \le y \le 1,\;t \le 0 \\ & t > 0:U = \gamma \frac{\partial u}{{\partial y}},\;\frac{\partial \theta }{{\partial y}} = Br[\theta - \theta_{a} ],\;at\;y = 0 \\ & U = 0,\;\theta = 0_{0} ,\;as\;y = 1 \\ \end{aligned}$$

### Steady state

8 $$\frac{{d^{2} U}}{{dy^{2} }} - \left( {H^{2} a + \frac{1}{Da}} \right)U + \theta = 0$$

9 $$\frac{{d^{2} \theta }}{{dy^{2} }} + \lambda e^{{\frac{\theta }{1 + \varepsilon \theta }}} = 0$$

The operating conditions that explain velocity slip and temperature jump situations at the fluid-wall interaction are as follows:

10 $$\begin{aligned} & U - \gamma \frac{dU}{{dy}} = 0,\quad \frac{d\theta }{{dy}} - Br[\theta - \theta_{a} ] = 0,\quad at\quad y = 0 \\ & U = 0,\quad \theta = 0,\quad at\quad y = 1 \\ \end{aligned}$$

## Method of solution

**Figure 1:**
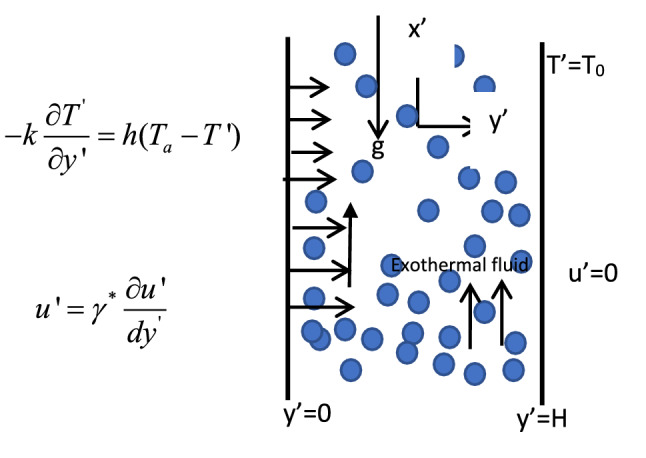
Physical coordinate of the flow system.

To solve the governing equation formulated from Fig. 1, we use the Homotopy perturbation approach to build a uniform Homotopy on Eqs. (5) and (6).

11 $$H(U,p) = (1 - p)\frac{{d^{2} U}}{{dy^{2} }} - p\left[ {\left( {H^{2} a + \frac{1}{Da}} \right)U - \theta } \right] = 0$$

12 $$H(\theta ,p) = (1 - p)\frac{{d^{2} \theta }}{{dy^{2} }} + p\left[ {\lambda e^{{\frac{\theta }{1 + \varepsilon \theta }}} } \right] = 0$$

We build Homotopy on Eqs. (4) and (5), supposing the resolution is in the form of:

13 $$\begin{aligned} \theta (Y) & = \theta_{0} + p\theta_{1} + p^{2} \theta_{2} + \cdots \\ U(Y) & = u_{0} + pu_{1} + p^{2} u_{2} + \cdots \\ \end{aligned}$$

Therefore, the following differential equations and their related revised boundary conditions may be obtained by inserting Eq. (6) into Eqs. (4) and (5) and contrast the coefficient of equal powers, p.

14 $$p^{0} :\;\frac{{d^{2} \theta_{0} }}{{dy^{2} }} = 0$$

15 $$\frac{{d^{2} u_{0} }}{{dy^{2} }} = 0$$

The changed matching boundary conditions are now

16 $$\left. \begin{gathered} \frac{{d\theta_{0} }}{dy} - Br[\theta_{0} - \theta_{a} ] = 0 \hfill \\ u_{0} - \gamma \frac{{du_{0} }}{dy} = 0 \hfill \\ \end{gathered} \right\}\;at\;y = 0$$

17 $$\left. \begin{gathered} \theta_{0} = 0 \hfill \\ u_{0} = 0 \hfill \\ \end{gathered} \right\}\;at\;y = 1$$

18 $$p^{1} :\;\frac{{d^{2} \theta_{1} }}{{dy^{2} }} + \lambda (1 + \theta_{0} + \left( {2 - \varepsilon } \right)\theta_{0}^{2} ) = 0$$

19 $$\frac{{d^{2} u_{1} }}{{dy^{2} }} - \left( {H^{2} a + \frac{1}{Da}} \right)u_{0} + \theta_{0} = A$$

This means that the associated boundary conditions have been transformed.

20 $$\left. \begin{gathered} \frac{{d\theta_{1} }}{dY} = Br[\theta_{1} ] \hfill \\ u_{1} = \gamma \frac{{du_{1} }}{dY} \hfill \\ \end{gathered} \right\}\;at\;y = 0$$

21 $$\left. \begin{gathered} \theta_{1} = 0 \hfill \\ u_{1} = 0 \hfill \\ \end{gathered} \right\}at\;y = 1$$

22 $$p^{2} :\;\frac{{d^{2} \theta_{2} }}{{dy^{2} }} + \lambda (\theta_{1} - 2\varepsilon \theta_{1} \theta_{0} + 4\theta_{1} \theta_{0} ) = 0$$

23 $$\frac{{d^{2} u_{2} }}{{dy^{2} }} - \left( {H^{2} a + \frac{1}{Da}} \right)u_{1} + \theta_{1} = 0$$

As a result, the changed boundary conditions are now

24 $$\left. \begin{gathered} \frac{{d\theta_{2} }}{dY} = Br[\theta_{2} ] \hfill \\ u_{2} = \gamma \frac{{du_{2} }}{dY} \hfill \\ \end{gathered} \right\}\;at\;y = 0$$

25 $$\left. \begin{gathered} \theta_{2} = 0 \hfill \\ u_{2} = 0 \hfill \\ \end{gathered} \right\}\;at\;y = 1$$

When *p* = 1, the approximated solutions of the differential equations yield

26 $$\begin{aligned} \theta& = \mathop {\lim \theta }\limits_{p \to 1} = \theta_{0} + \theta_{1} + \theta_{2} + \cdots \\ U& = \mathop {\lim U}\limits_{p \to 1} = u_{0} + u_{1} + u_{2} + \cdots \\ \end{aligned}$$

Some terms can be approximated by the trend (19) in most circumstances. The converges rate is determined by the nonlinear operator. When using the Homotopy perturbation approach to arrive at the series solution, only a few terms from the HPM sequence might be employed to approximate the result. The following are the responses to: $\theta_{0} ,\theta_{1} , \ldots \;and\;u_{0} ,u_{1} ,u_{2} ...$.

27 $$\theta_{0} = C_{0} + C_{1} y$$

28 $$\theta_{1} = - \lambda \left[ {\frac{{y^{2} }}{2} + C_{0} \frac{{y^{3} }}{6} + C_{1} \frac{{y^{2} }}{2} + Q\left( {C_{0}^{2} \frac{{y^{4} }}{12} + C_{0} C_{1} \frac{{y^{3} }}{3} + C_{1}^{2} \frac{{y^{2} }}{2}} \right)} \right] + C_{2} y + C_{3}$$

29 $$\theta_{2} = - (4\lambda - 2\varepsilon \lambda )\left[ {\lambda \left\{ {\frac{{y^{4} }}{24} + C_{0} \frac{{y^{5} }}{120} + C_{1} \frac{{y^{4} }}{24} + Q\left( {C_{0}^{2} \frac{{y^{6} }}{360} + C_{0} C_{1} \frac{{y^{5} }}{60} + C_{1}^{2} \frac{{y^{4} }}{24}} \right)} \right\}} \right. + C_{2} \frac{{y^{3} }}{6} + C_{3} \frac{{y^{2} }}{2} + C_{4} y + C_{5}$$

30 $$u_{1} = - \left[ {C_{0} \frac{{y^{3} }}{6} + C_{1} \frac{{y^{2} }}{2}} \right] + C_{8} y + C_{9}$$

31 $$u_{2} = a_{2} \left( { - C_{0} \frac{{y^{5} }}{120} - C_{1} \frac{{y^{4} }}{24} + C_{8} \frac{{y^{3} }}{6} + C_{9} \frac{{y^{2} }}{2}} \right) + \lambda \left( {\frac{{y^{4} }}{24} + C_{0} \frac{{y^{5} }}{120} + C_{1} \frac{{y^{4} }}{24} + Q\left( {C_{0} \frac{{y^{6} }}{360} + C_{0} C_{1} \frac{{y^{5} }}{60} + C_{1}^{2} \frac{{y^{4} }}{24}} \right)} \right) - C_{2} \frac{{y^{3} }}{6} - C_{3} \frac{{y^{2} }}{2} + C_{10} y + c_{11}$$

The skin friction and rate of heat transfer over both surfaces are calculated by differentiating the temperature and velocity equations with respects to y.

32 $$Nu_{0} \Leftrightarrow \left. {\frac{d\theta }{{dy}}} \right|_{y = 0} = C_{0} + C_{2} + C_{4}$$

33 $$\begin{aligned} & Nu_{1} \Leftrightarrow \left. {\frac{d\theta }{{dy}}} \right|_{y = 1} = C_{0} - \lambda \left[ {1 + \frac{{C_{0} }}{2} + C_{1} + Q(C_{0} C_{1} + C_{1} )} \right] + C_{2} \\ & \quad - (4\lambda - 2\varepsilon \lambda )\left[ {\left[ {\frac{{C_{0} }}{6}} \right. + \frac{{C_{0}^{2} }}{6} + \frac{{C_{0} C_{1} }}{24} + Q( + \frac{{C_{0}^{2} }}{60} + \frac{{C_{0} C_{1} }}{12} + \frac{{C_{1}^{2} }}{6})} \right] + \frac{{C_{2} }}{2} + C_{3} + C_{4} \\ \end{aligned}$$

34 $$Sk_{0} \Leftrightarrow \left. {\frac{du}{{dy}}} \right|_{y = 0} = C_{8} + C_{10}$$

35 $$\begin{aligned} & Sk_{1} \Leftrightarrow \left. {\frac{du}{{dy}}} \right|_{y = 1} = \left( {\frac{{C_{0} }}{2} + C_{1} } \right) + C_{8} + a_{2} \left( {\frac{{C_{0} }}{24} + \frac{{C_{1} }}{6} + \frac{{C_{8} }}{2} + C_{9} } \right) \\ & \quad + \left( { - \lambda (\frac{1}{24} + \frac{{C_{0} }}{24} + \frac{{C_{1} }}{6} + Q(\frac{{C_{0}^{2} }}{60} + \frac{{C_{0} C_{1} }}{12} + \frac{{C_{1}^{2} }}{6} + \frac{{C_{2} }}{2} + C_{3} ))} \right) + C_{10} \\ \end{aligned}$$

The constant $C_{0} ,C_{1} ,C_{3} ,C_{4} ,C_{5} ,C_{6} ,C_{7} ,C_{8} ,C_{9} ,C_{10} ,\,Q$ can be found in Appendix.

## Numerical solution

The implicit finite difference scheme is used to solve a set of partial differential equations with boundary conditions. For all time dependencies, we used the forward difference formula and approximated the first and second derivatives with second order central differences. The last nodes are adjusted to reflect the boundary conditions, and the equations correspond to the first derivatives. Equations (5) and (6) have an implicit finite difference equation, which is as described in the following:

36 $$- rU_{i - 1}^{j - 1} + (1 + 2r)U_{i}^{j + 1} - rU_{i + 1}^{j + 1} = (1 - M^{2} )U_{i}^{j} + \left[ {1 - M^{2} \Delta t - \frac{1}{Da}\Delta t} \right]U_{i}^{j}$$

37 $$- r\theta_{i - 1}^{j - 1} + (\Pr + 2r)\theta_{i}^{j + 1} - r\theta_{i + 1}^{j + 1} = \Pr \theta_{i}^{j} + \lambda \Delta t\exp \left( {\frac{{\theta_{i}^{j} }}{{1 + \varepsilon \theta_{i}^{j} }}} \right)$$

## Discussion of findings

**Figure 2:**
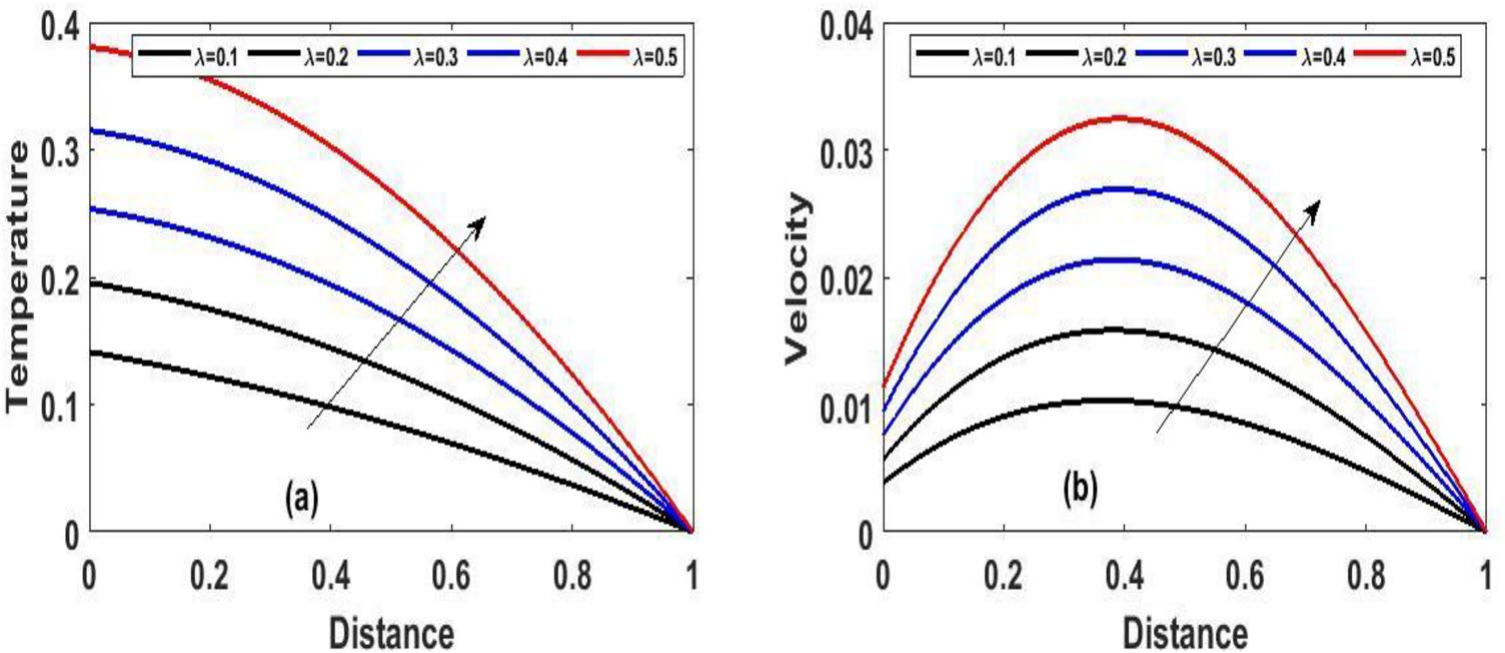
The effect of variation of $(\lambda )$ for Temperature and velocity steady state.

**Figure 3:**
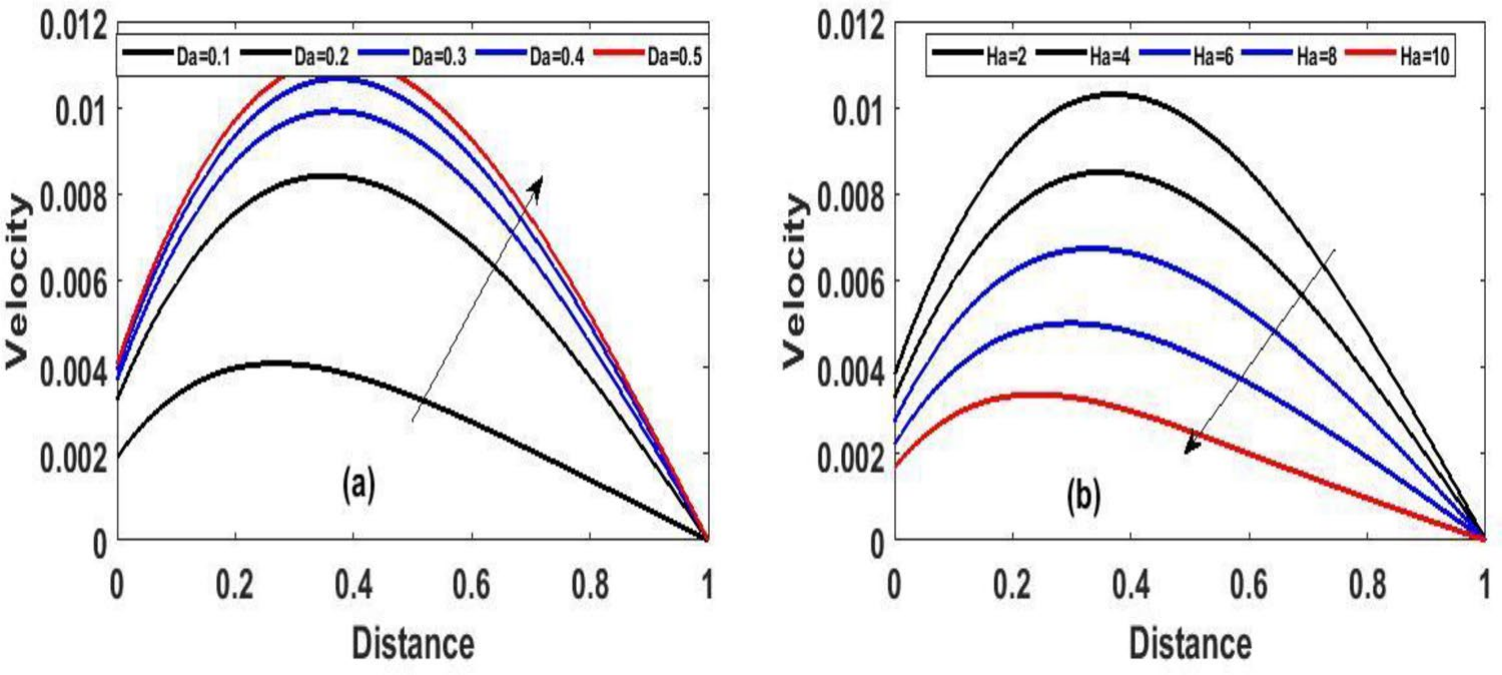
The effect of variation of $Da$ and $Ha$ for velocity steady state.

**Figure 4:**
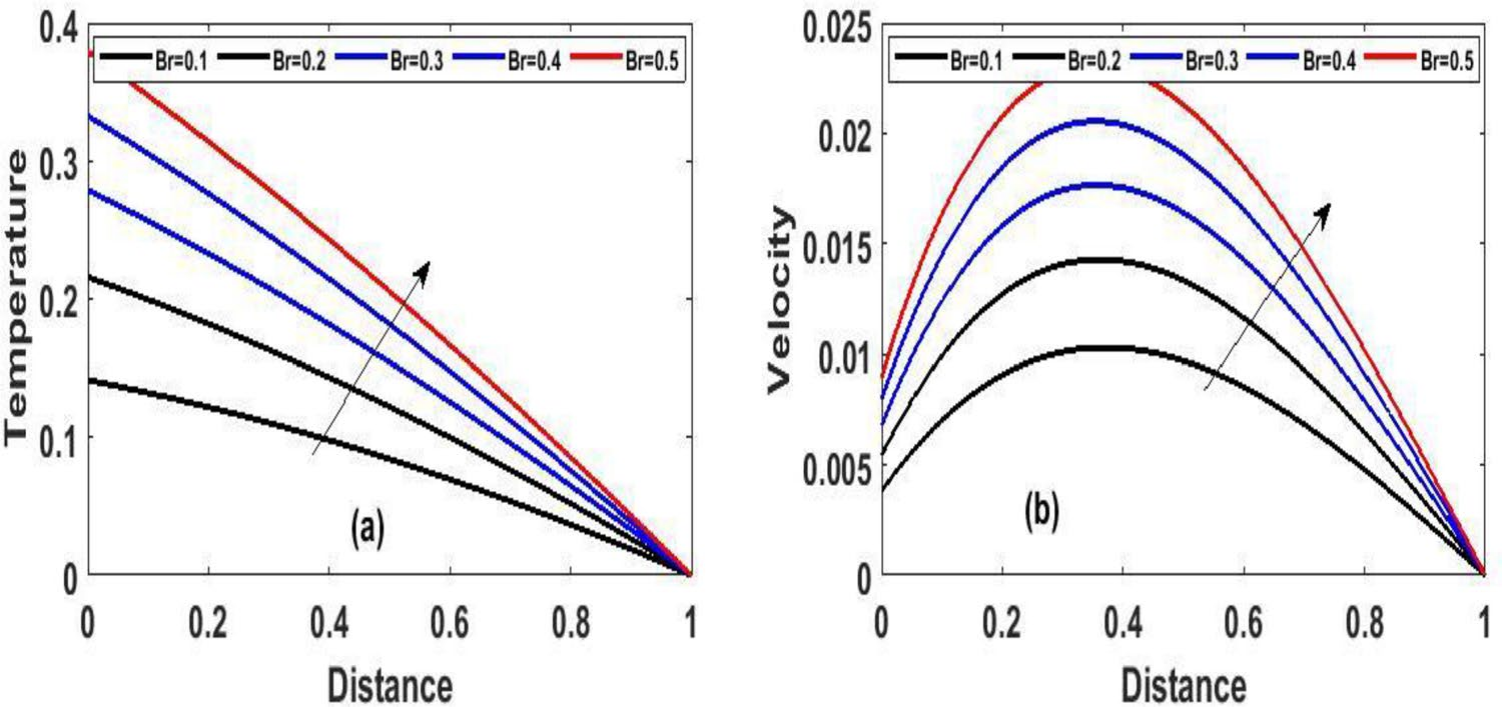
The effect of variation of $Br$ for Temperature and velocity steady state.

**Figure 5:**
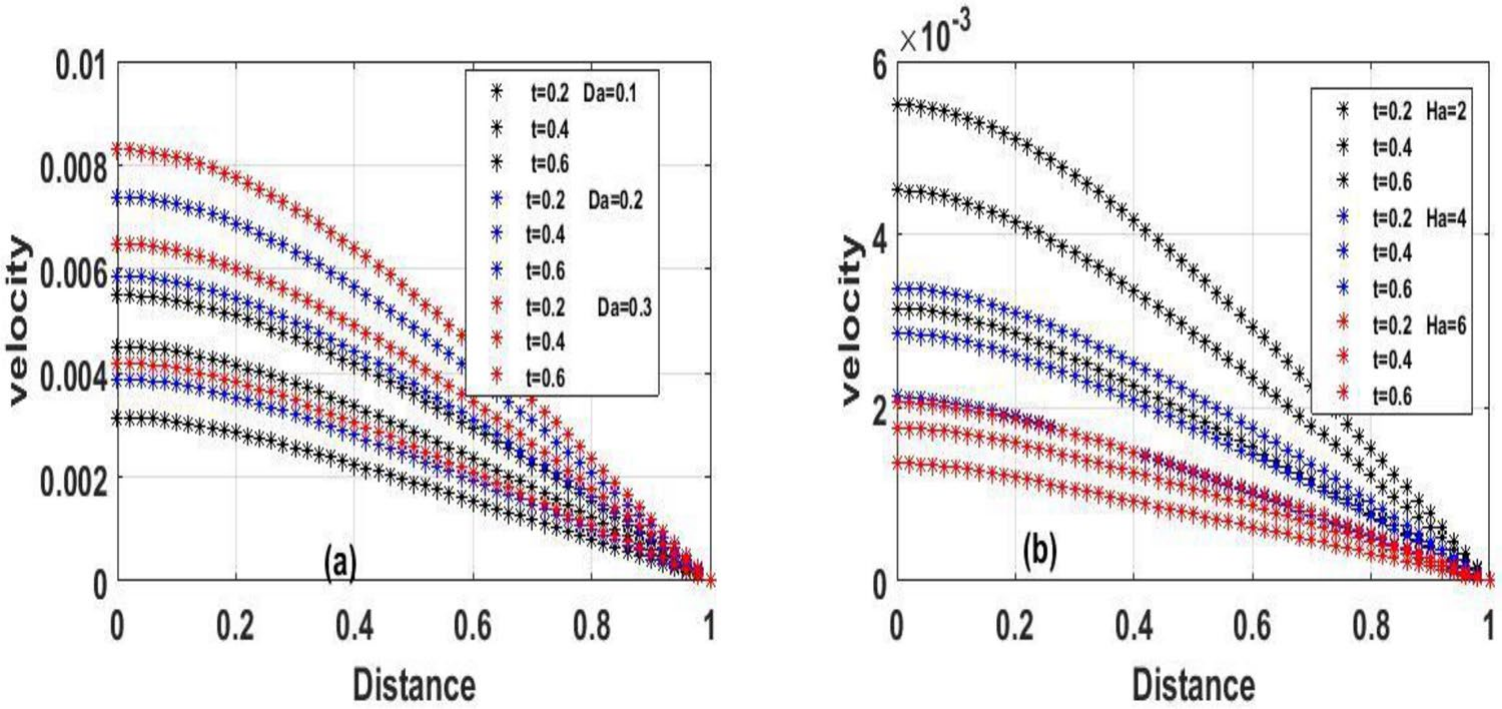
The effect of variation of $Da\,\,$ and $Ha$ for velocity unsteady state.

**Figure 6:**
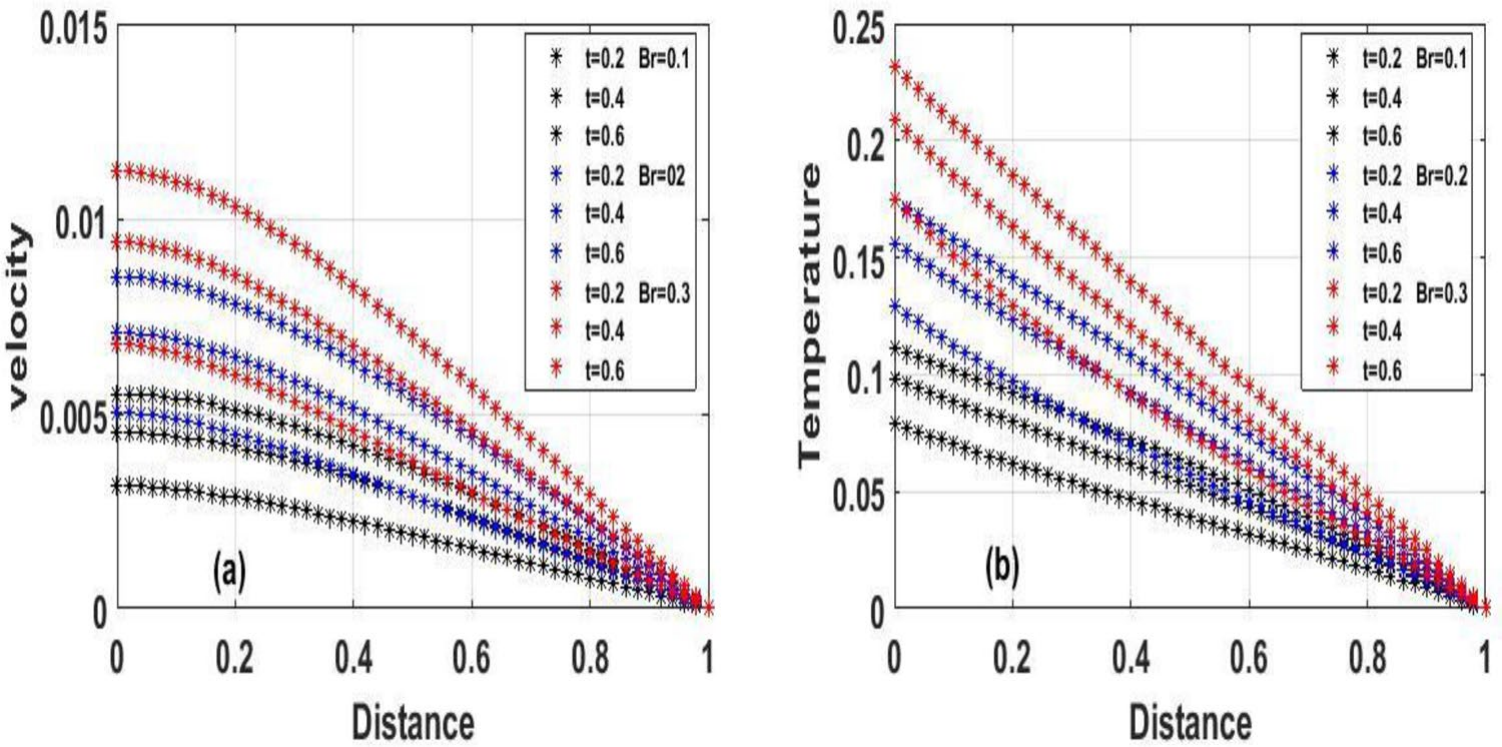
The effect of variation of $Br$ for Temperature and velocity unsteady state.

**Figure 7:**
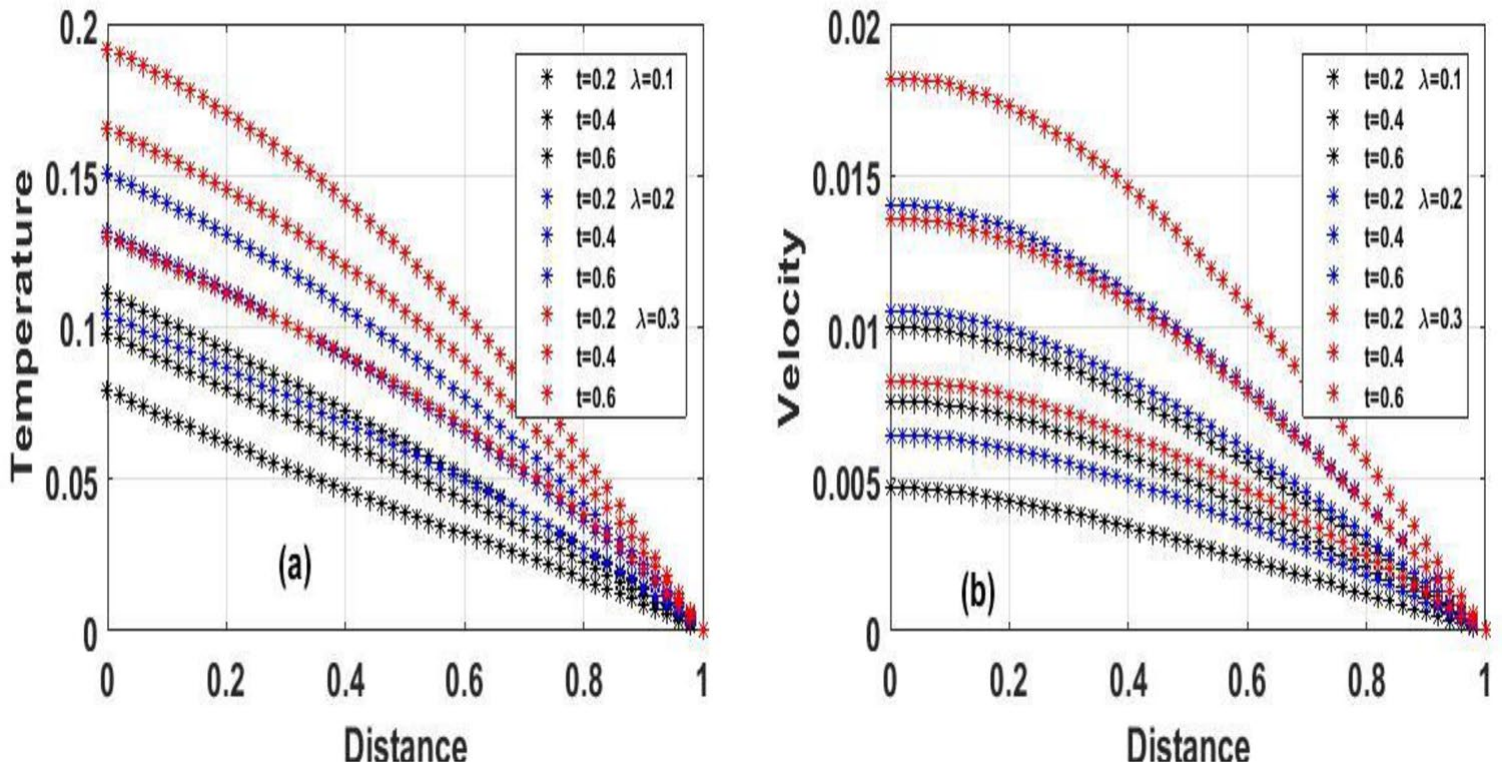
The effect of variation of $\lambda$ for Temperature and velocity unsteady state.

**Figure 8:**
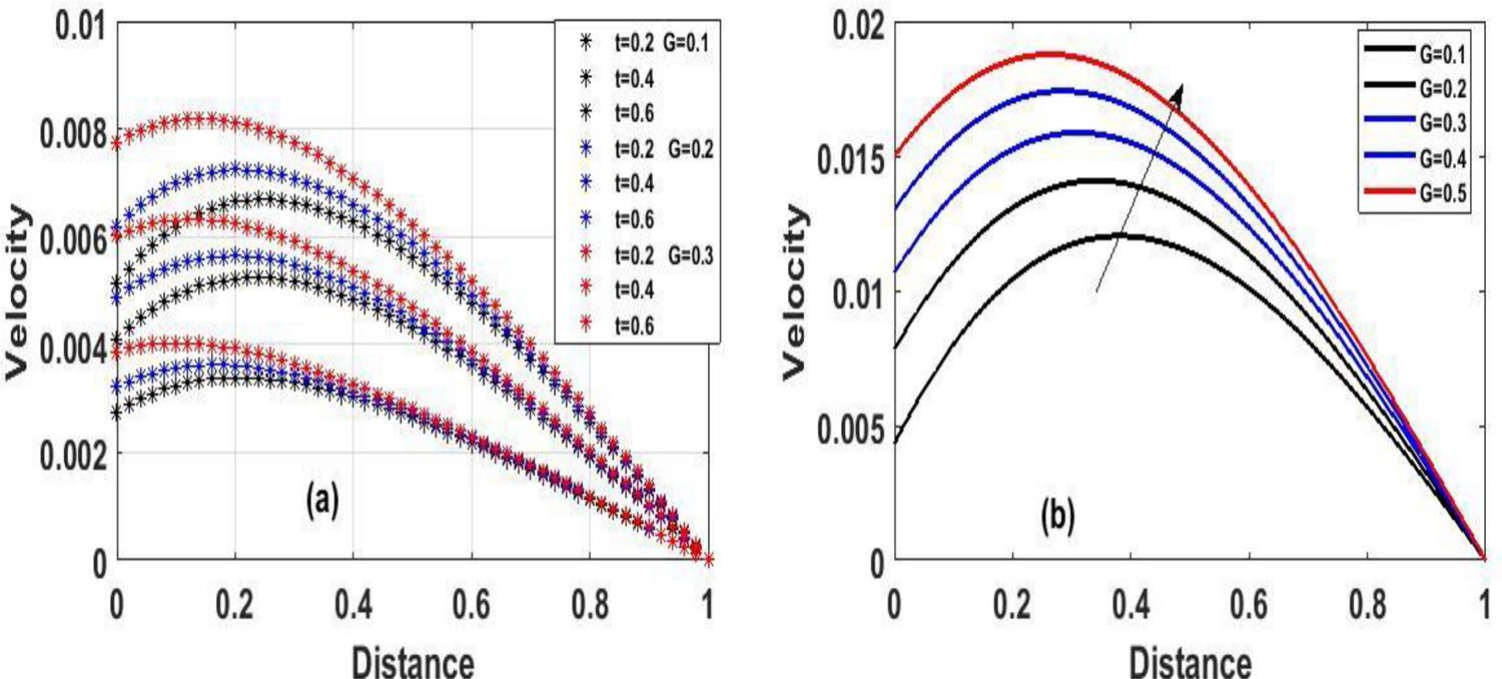
The effect of variation of $\gamma$ for velocity steady and unsteady state.

**Figure 9:**
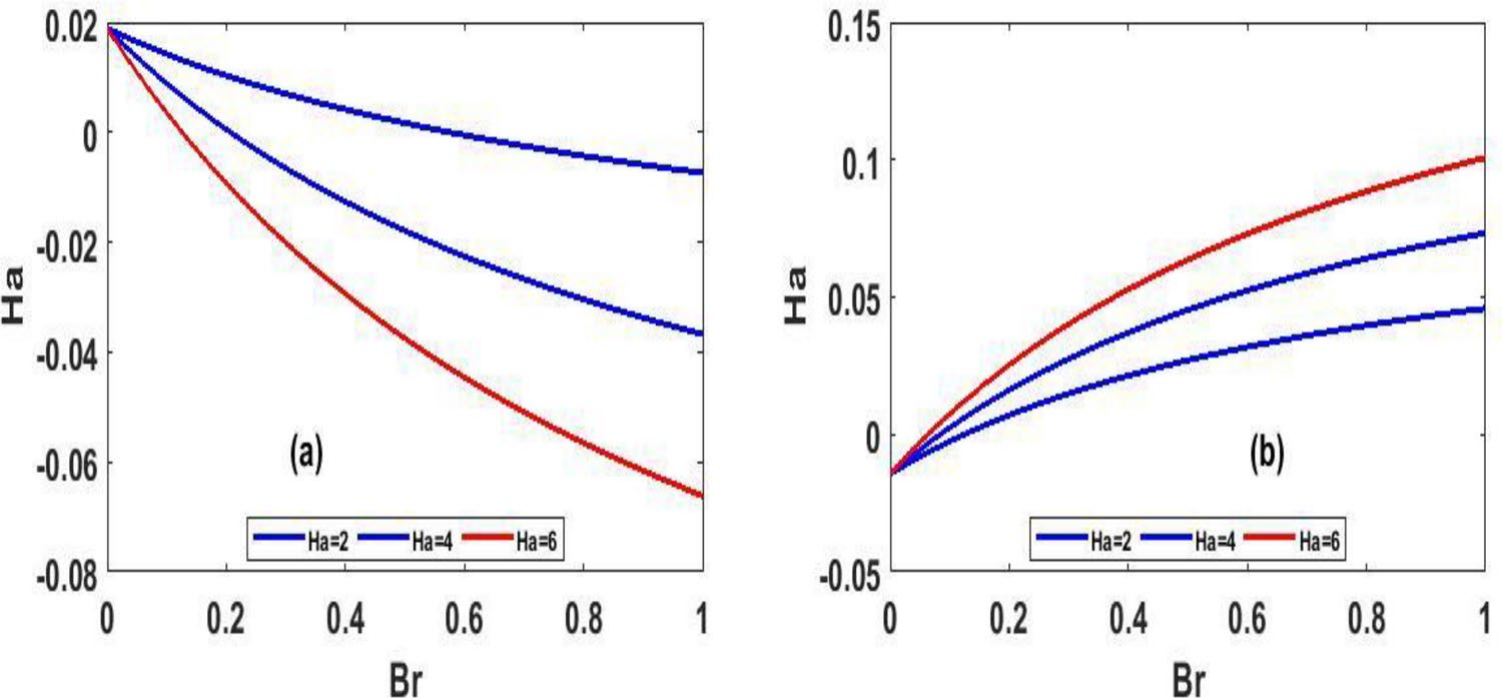
Steady state skin friction for $(Ha)$ against $(Br)$.

**Figure 10:**
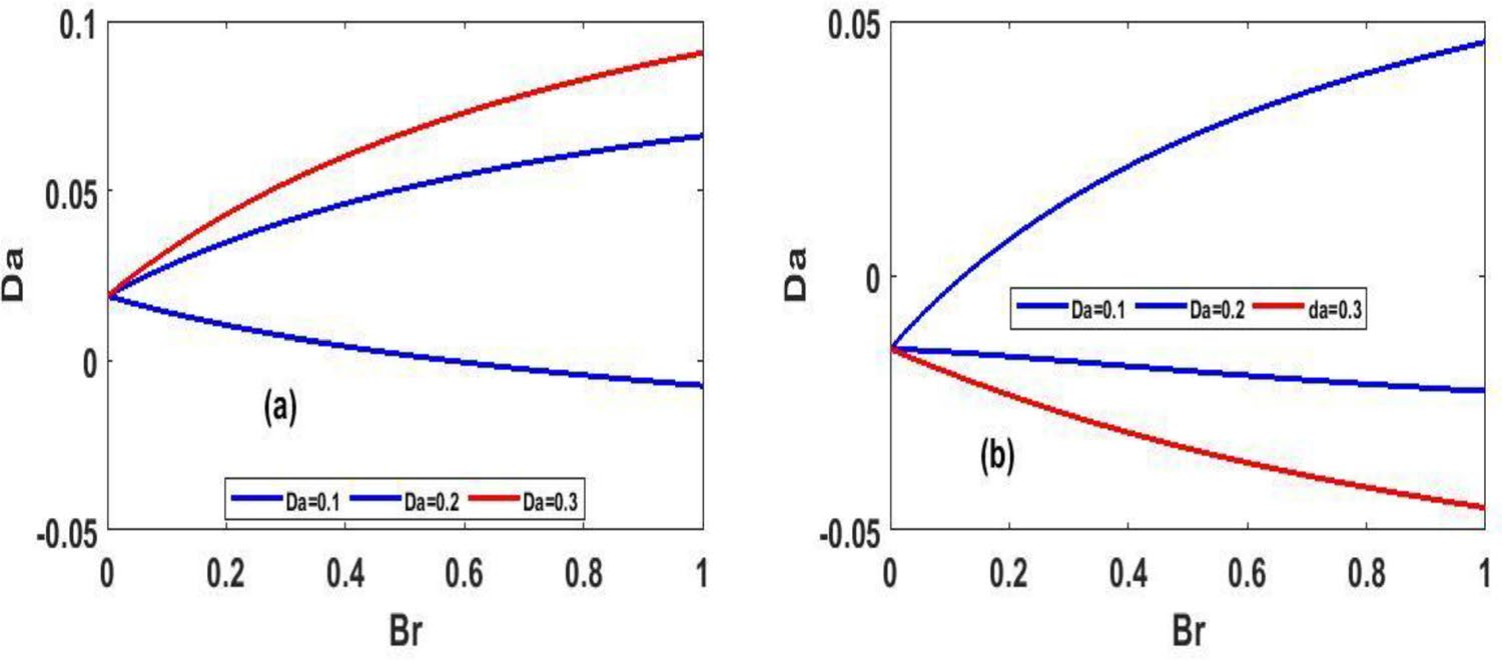
Steady state skin friction for $(Da)$ against $(Br)$.

**Figure 11:**
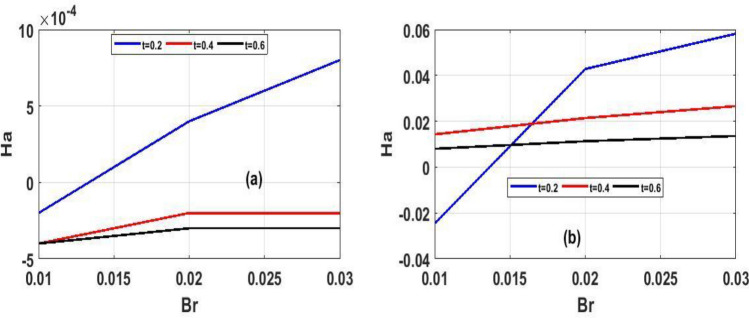
Unsteady state skin friction for $(Ha)$ in proportion to $(t)$.

**Figure 12:**
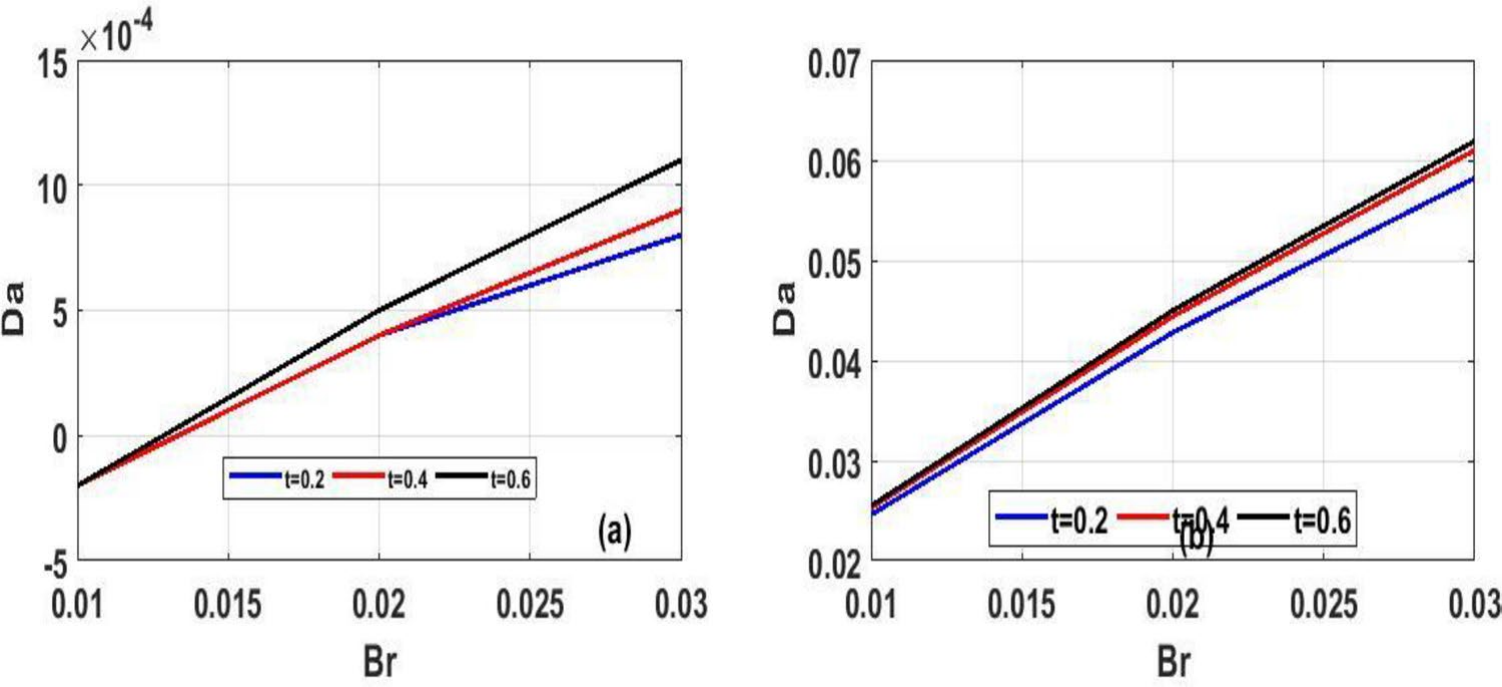
Unsteady skin friction for $(Da)$ in proportion to $(t)$.

**Figure 13:**
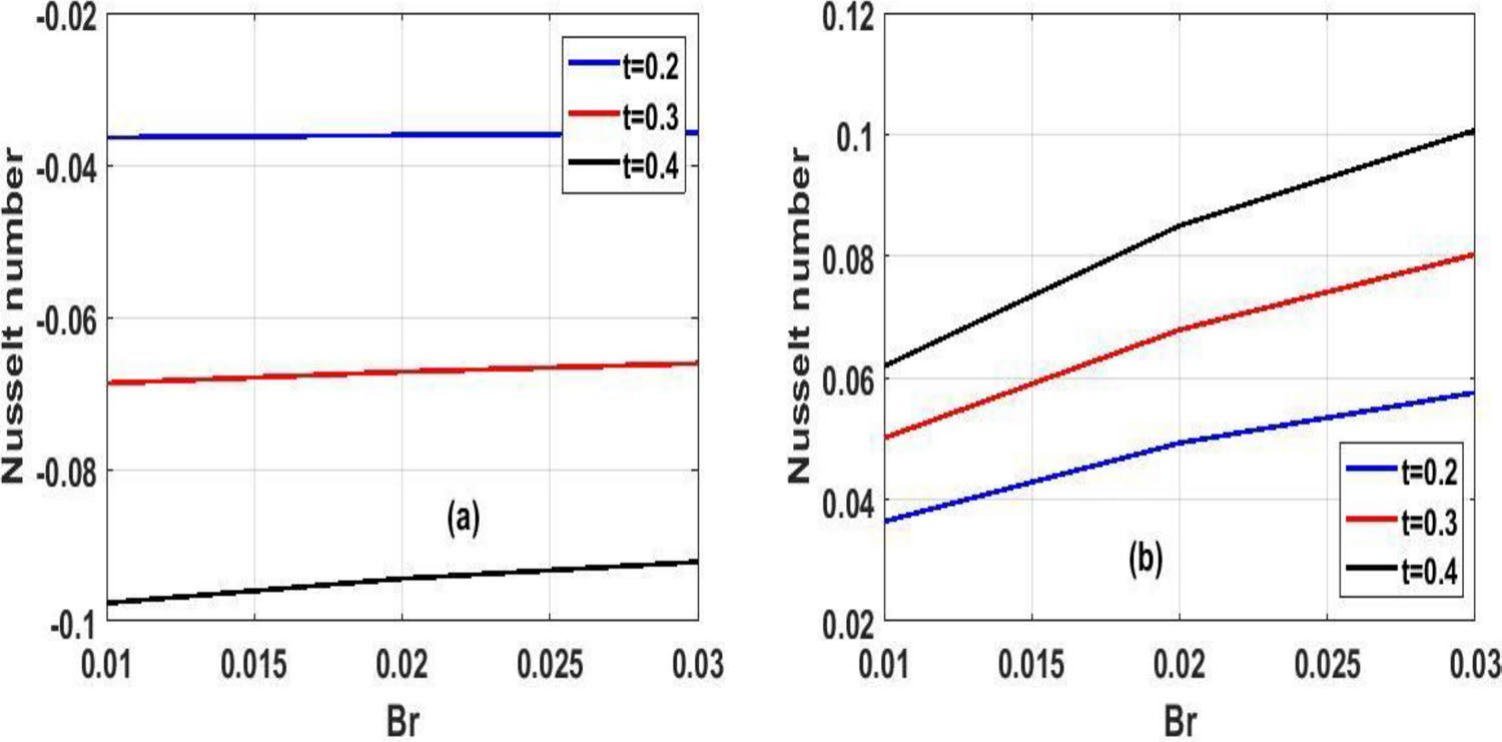
Unsteady Nusselt number for $(\lambda )$ in proportion to $(t)$.

**Figure 14:**
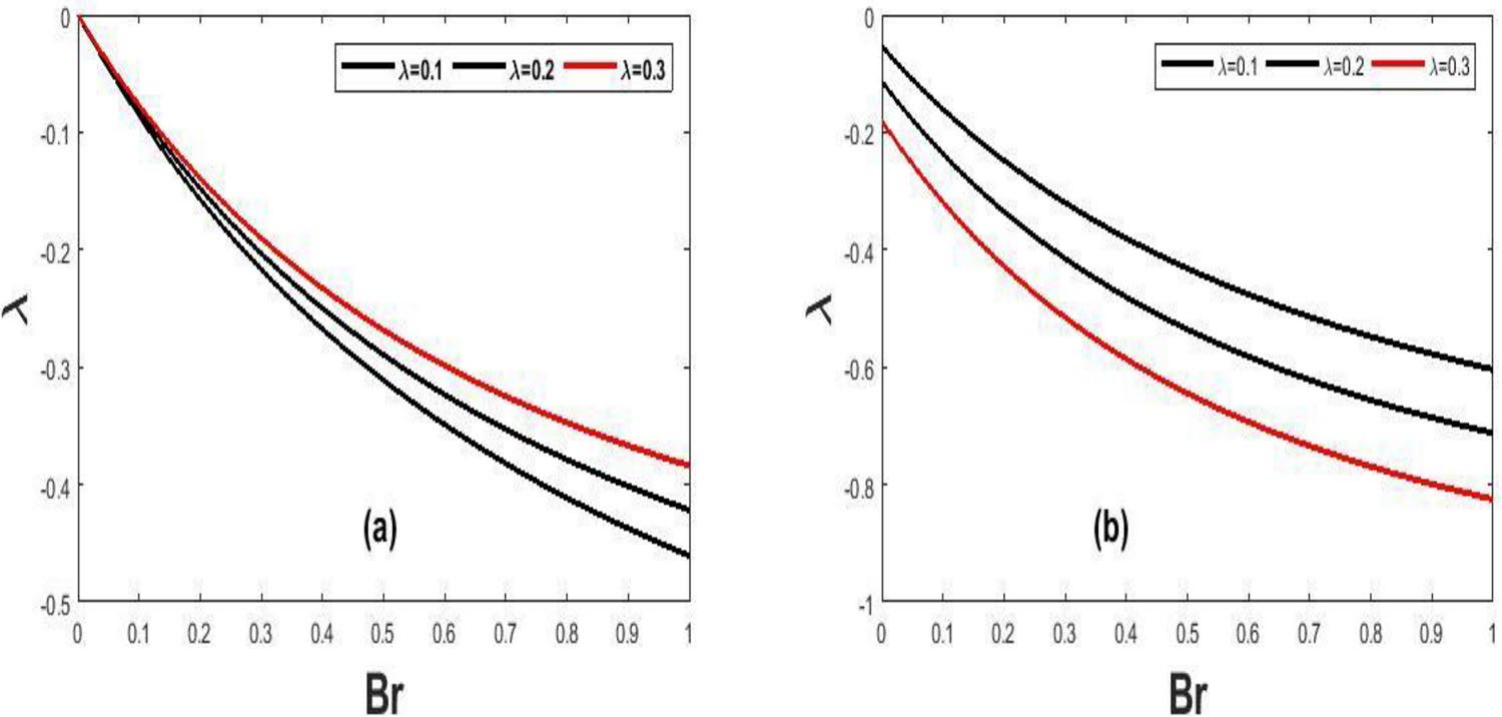
Steady state Nusselt number for $(Br)$ against $(\lambda )$.

The steady state solution of Eqs. (8) and (9) with respect to the boundary condition (10) were solved using one of the most efficient method called Homotopy perturbation method, however, we employed implicit finite difference scheme to address Eqs. (5) and (6) with respect to the boundary condition (7). The effects of a various involving parameters on fluid flow, temperature, velocity, skin friction coefficient, and heat transfer rate are graphically examined. Unless otherwise indicated, the reactive viscous parameter's impacts like Frank-Kamenetskii ($\lambda$), Hartman number $(Ha^{2} )$, Navier slip parameter ($\gamma$), Darcy number $(Da)$ and local Biot number $(Br)$ in the fluid flow were verified in Figs. 2, 3, 4, 5, 6, 7, 8, 9, 10, 11, 12, 13, 14. The numerical values for the parameters are assumed to be $Ha^{2} = 2,\,\,\lambda = 0.1,\gamma = 0.1,\,\,Br = 0.1,\,\,Da = 0.1,\,\,\,\Pr = 0.71,\,\,e = 0.1$.The period intervals utilized are $0.2 \le t \le 0.6$. The rationale behind choosing this value is that when the fluid pressure exceeds the set value, there is every tendency for the fluid to escape within the model.

Figure 2a and b Explain how the Frank-Kamenetskii parameter played a significat role in both energy and momentum profiles; it is clear that increasing values of $(\lambda )$ increase both the temperature and velocity of the fluid. Increasing the value of $(\lambda )$ strengthens the chemical reaction and viscous heating source factors in the temperature equation, resulting in a considerable temperature rise. As seen in Fig. 2a and b, as the substantial temperature rises in response to the increase in $(\lambda )$, the fluid viscosity decreases, resulting in an increase in fluid velocity. In addition, the highest values of fluid temperature and velocity are reached at the bottom plate surface of the channel and decrease toward the top plate. This phenomenon is caused by asymmetric heating of the plate. The effects of porous medium $(Da)$ and $(Ha)$ are depicted in Fig. 3a and b, respectively. In Fig. 3a, the flow increased due to an increase in permeability, which resulted in an increase in fluid velocity. Essentially speaking, A solid or group of solids can be classified as a porous medium if there is enough free space inside or around the particles for a fluid to pass through. Oil, gas, and water flow are all examples of this. To name just a few areas where this technology may be used the food and beverage industry; petroleum technology; agricultural technology; biomedical technology; and geothermal technology. however, in Fig. 3b, the Hartman number was varied, Furthermore, it was found that a rise in Hartman induced the Lorentz force to appear, which facilitated the momentum boundary layer's flow, thereby retarding the fluid flow. Figure 4a and b show how the thermal Biot number $(Br)$ effects on temperature and velocity. When $(Br)$ is increased, the channel surface and inside temperature increase, as seen in Fig. 4a, b. This increases the thickness of the thermal boundary layer, as well as the temperature and velocity distribution of the flow, as the thermal Biot number rise. The effect of Darcy number and dimensionless time $(t)$ on the velocity profile is seen in Fig. 5a. The velocity of the fluid is increased when the Darcy number increases, as seen in the figure and the fluid velocity tends to increase as time passes. While the effects of $(Ha)$ on the flow were detailed in Fig. 5b, an increase $(Ha)$ results in the presence of a drag force known as the Lorentz force, which resists the fluid flow, resulting in a decrease in velocity fluid flow, then as time $(t)$ increases as the number of drag forces opposing the fluid flow decreases resulting to increase in fluid flow.

The effect of the local Biot number $(Br)$ in respect of dimensional $(t)$ time is explored in Fig.6a and b. A slight increase in $(Br)$ corresponds to increase in fluid momentum and energy at the bottom surface channel. Because high numbers of local Biot number $(Br)$ denoted a greater degree of convective heating which means as time $(t)$ passes and $(Br)$ increases, so does the energy and momentum fluid flow increases. From a critical standpoint, Newtonian heating is a process in which internal resistance is minimal relative to surface resistance; its applications comprise heat exchangers, bidirectional heat transfer over fins, the fossil fuel industry, and solar radiation.

Figure 7a and b show the effect of viscous reactive fluid on transient velocity and temperature profiles, with a slight uptick in $(\lambda )$ resulting to a considerable increase in fluid flow. This is due to the energy equation's viscous heating, which causes a temperature spike, resulting in a decrease in fluid viscosity which resulted to an increase in fluid velocity and temperature, however, as time $(t)$ increase the fluid state also increase. Figure 8a and b potrayed varying of (γ)﻿ on both steady and unsteady state velocity, an increase in (γ)﻿ result to an increase in fluid flow due to the fact that greater $(\gamma )$ values tend to boost responsiveness and slickness at the bottom plate, and as time $(t)$ passes by, the fluid velocity continues to rise in Fig. 8a. However, Figure 8b shows that $(\gamma )$ increases in velocity as a result of the bottom plate's responsiveness and slickness.

The variation of steady state skin friction $(Ha)$ against $(Br)$ is shown in Fig. 9a and b respectively, It is revealed from the profile that increases in Hartmann number decreases the skin friction primarily due to decrease in the boundary layer thickness. Steady state skin friction was elucidated in Fig. 10a and b respectively, as $(Da)$ increase the skin friction increase Fig. 10a while reverse phenomenon is seen in Fig. 10b. Figure 11a and b portrayed the variation of skin friction $(Ha)$ against dimensional time $(t)$. It is observed that an increase in magnetic fluid decreases the skin friction at lower plate and also as time $(t)$ increases the skin friction also decrease in Fig. 11b and b respectively this is due to the drag occasioned by the effect of the Lorentz force on the flow. The skin friction increases in both plate of Fig.12a and b with increase in porous medium $(Da)$ and also as time $(t)$ increase skin friction also increase. Figure 13a and b demonstrate the rate of heat transfer local Biot number $(Br)$ over dimensionless time $(t)$ a higher number of local Biot number $(Br)$. equates to a high rate of heat transfer at the upper plate of both Figs. 13a and b. the rate of heat transfer for steady state is illustrated in Fig. 14a and b Nusselt number increase in 14a while decrease in 14b. A comparison of velocity profiles in Table 1 demonstrates strong agreement between the results of Reference ^32^ and our work, but the absence of Hartmann and Darcy values from Table 2 depicted good agreement between our findings and that of Reference ^32^.

**Table 1:** Shows numerical computations comparing Reference^32^ work to the present work for velocity profiles with varying value of $y$.

$(y)$	Reference^32^ $U(y)$	Present work $U(y)$
0.1	0.0184	0.0182
0.2	0.0148	0.0146
0.3	0.0112	0.0110
0.4	0.0075	0.0074
0.5	0..0038	0.0037

$$\gamma = 0.1,\;\lambda = 0.1$$ $$Da = 100000,\;Br = 0.1$$ $$Ha = 0.$$

**Table 2:** Comparison of skin-frictions by neglecting Hartmann number and Darcy number.

$Br$	$\tau_{0}$		$\tau_{1}$	
	Reference^32^	Present results	Reference^32^	Present results
0.1	0.31098	0.31093	− 0.00396	− 0.00391
0.2	0.28231	0.28227	− 0.00343	0.00339
0.3	0.24339	0.24336	0.00315	0.00311
0.4	0.20147	0.20145	0.00276	0.00273
0.5	0.16491	0.16489	0.00220	0.00218

$$\gamma = 0.1,\;\lambda = 0.1$$ $$Da = 100000$$, $$Ha = 0.$$

## Conclusion

Mathematical model has been developed and solved exactly to analyze the impact of magnetohydrodynamic on unsteady fluid flow of an exothermic reaction in a vertical channel with distance H. The Navier slip and Newtonian heating of the channel are incorporated in the mathematical model. We applied Homotopy perturbation method for steady state governing equation while the unsteady state governing equation were solved by Implicit finite different scheme technique. However, by solving the current mathematical model, equations for velocity, temperature, rate of heat transfer as well as skin friction coefficient were derived. The following are the key findings of this study:

1. It was noticed that, velocity increase with increasing values of darcy number $(Da)$.
2. Velocity profile experience significant surge as increase in Navier slip $(\gamma )$., local biot number $(Br)$..and viscous reactive fluid parameter $(\lambda )$.
3. It was also revealed that velocity profile decreases for an increase in magnetic field parameter $(Ha)$.
4. The action of Hartmann number decreases skin friction coefficients on the surface of y = 0 but converse on the surface of y = 1.
5. it was depicted that the behavior of porous medium parameter increases skin friction on both channels, where the fluid flow is higher at upper channel compare to bottom channel.

## Supplementary Information

Supplementary Information.

## Data Availability

The datasets used and/or analyzed during the current study available from the corresponding author on reasonable request.
