# Supplementary material for: Unsteady MHD free convection flow of an exothermic fluid in a convectively heated vertical channel filled with porous medium
Source: Sci Rep. 2022 Jul 14;12:11989. doi: 10.1038/s41598-022-16064-y (PMC9283519; doi:10.1038/s41598-022-16064-y)
Supplement: Supplementary file 1 — Supplementary Information. [file 41598_2022_16064_MOESM1_ESM.docx]

**Appendix**

$$\begin{matrix} k_{0}=\frac{Br\theta a}{1+Br}, k_{1}=-k_{0}, Q=2-\varepsilon, k_{2}=Brk_{3} \\ \\ k_{3}=\frac{\lambda\left[\frac{1}{2}+\frac{k_{0}}{6}+\frac{k_{1}}{2}+Q(\frac{k_{0}^{2}}{12}+\frac{k_{0}k_{1}}{3}+\frac{k_{1}^{2}}{2})\right]}{\left[1+Br\right]}, \\ \\ k_{5}=\frac{\lambda\left[-\lambda\left\{\frac{1}{24}+\frac{k_{0}}{120}+\frac{k_{1}}{24}+Q(\frac{k_{0}^{2}}{360}+\frac{k_{0}k_{1}}{60}+\frac{k_{1}^{2}}{24})\right\}\right]+\frac{k_{2}}{6}+\frac{k_{3}}{2}}{\left[1+Br\right]}, \\ \\ k_{4}=Brk_{5} k_{6}=\gamma k_{7}, k_{7}=\frac{\frac{Gr}{\mathrm{Re}}\left[\frac{k_{0}}{6}+\frac{k_{1}}{2}\right]-\frac{A}{2}}{\left[1+\gamma\right]}, \\ k_{8}=\gamma k_{9}, \\ \\ k_{9}=\frac{Ha^{2}\left[\frac{-Gr}{\mathrm{Re}}\left(\frac{k_{0}}{120}+\frac{k_{1}}{24}\right)+\frac{A}{24}+\frac{k_{8}}{6}+\frac{k_{9}}{2}\right]-\frac{Gr}{\mathrm{Re}}\left[-\lambda\left(\frac{1}{24}+\frac{k_{0}}{120}+\frac{k_{1}}{24}+Q\left(\frac{k_{0}}{360}+\frac{k_{0}k_{1}}{60}+\frac{k_{1}^{2}}{24}\right)+\frac{k_{2}}{6}+\frac{k_{3}}{2}\right)\right]}{1+\gamma} \end{matrix}$$

**Nomenclature**

 acceleration due to gravity universal gas constant

 Prandtl number ambient temperature

 dimensionless distance between the plates thermal conductivity of the fluid

 dimensional time activation energy

 dimensionless time heat transfer coefficient

 dimensional temperature of the fluid Greek letters

 initial temperature of the fluid and plates $\beta$ volumetric coefficient of thermal expansion

 initial concentration of the fluid and plates $\lambda$ Frank- Kamenetskii parameter

 dimensional velocity of the fluid dimensionless temperature

 dimensionless velocity of the fluid $\rho$ density of the fluid

 specific heat of the fluid at constant pressure $\varepsilon$ activation energy parameter

 initial concentration of the reactant species $\kappa$ dimensionless slip velocity

 dimensional coordinate parallel to the plate $\nu$ kinematic viscosity

 dimensional coordinate perpendicular to the plate

 dimensional coordinate perpendicular to the plate
